# Sleeping and Eating Behavior Recognition of Horses Based on an Improved SlowFast Network

**DOI:** 10.3390/s24237791

**Published:** 2024-12-05

**Authors:** Yanhong Liu, Fang Zhou, Wenxin Zheng, Tao Bai, Xinwen Chen, Leifeng Guo

**Affiliations:** 1 College of Computer and Information Engineering, Xinjiang Agricultural University, Urumqi 830052, China; lzyfhf@gmail.com (Y.L.); bt@xjau.edu.cn (T.B.); 2 Agricultural Information Institute, Chinese Academy of Agricultural Sciences, Beijing 100080, China; 3 Xinjiang Agricultural Informatization Engineering Technology Research Center, Urumqi 830052, China; 4 Ministry of Education Engineering Research Centre for Intelligent Agriculture, Urumqi 830052, China; 5 College of Information Science and Technology, Shihezi University, Shihezi 832000, China; zf_shzu@shzu.edu.cn; 6 Institute of Animal Husbandry Quality Standards, Xinjiang Academy of Animal Science, Urumqi 830011, China; zwx2020@126.com

**Keywords:** horse, computer vision, body posture detection, behavior recognition, improved SlowFast network

## Abstract

The sleeping and eating behaviors of horses are important indicators of their health. With the development of the modern equine industry, timely monitoring and analysis of these behaviors can provide valuable data for assessing the physiological state of horses. To recognize horse behaviors in stalls, this study builds on the SlowFast algorithm, introducing a novel loss function to address data imbalance and integrating an SE attention module in the SlowFast algorithm’s slow pathway to enhance behavior recognition accuracy. Additionally, YOLOX is employed to replace the original target detection algorithm in the SlowFast network, reducing recognition time during the video analysis phase and improving detection efficiency. The improved SlowFast algorithm achieves automatic recognition of horse behaviors in stalls. The accuracy in identifying three postures—standing, sternal recumbency, and lateral recumbency—is 92.73%, 91.87%, and 92.58%, respectively. It also shows high accuracy in recognizing two behaviors—sleeping and eating—achieving 93.56% and 98.77%. The model’s best overall accuracy reaches 93.90%. Experiments show that the horse behavior recognition method based on the improved SlowFast algorithm proposed in this study is capable of accurately identifying horse behaviors in video data sequences, achieving recognition of multiple horses’ sleeping and eating behaviors. Additionally, this research provides data support for livestock managers in evaluating horse health conditions, contributing to advancements in modern intelligent horse breeding practices.

## 1. Introduction

Horses are common domestic animals, often used for sports, companionship, and various work roles [1]. Historically, horses were considered crucial partners in agriculture and warfare, especially in transportation. Today, they play prominent roles in sports, leisure, and tourism [2]. In the equine industry, particularly for sport horses, continuous individual stall housing is prevalent. However, increasing scientific evidence suggests that such stall conditions may have negative effects on the health of horses [3]. The behaviors of a horse are key indicators of their health status, as changes in behaviors can signal underlying health issues. Therefore, monitoring horse behaviors in stalls to assess their health status is important in modern equine management. However, there are various problems such as high cost, focus, and easy fatigue in monitoring multiple horses manually. Therefore, it is necessary to use advanced equipment and technical means to identify horse behaviors.

In recent years, researchers have explored various methods for animal behavior recognition. Some have focused on using intelligent wearable devices to identify animal behaviors and have achieved promising results. For instance, wearable devices have been used on sheep, employing traditional machine learning algorithms to classify their behaviors [4,5]. Additionally, when faced with predators like wild dogs, sheep exhibit a series of anti-predator behaviors. Researchers have utilized wearable smart sensors to analyze these behaviors, providing a comprehensive understanding of sheep’s responses to predators [6]. Wearable smart devices have also been applied to the behavior analysis of dairy cows, enabling the detection of abnormal behaviors in sick cows [7] as well as the analysis of common behaviors in cows [8]. Furthermore, monitoring milk yield is a critical aspect for dairy farmers, and the use of intelligent wearable devices for analyzing milk production [9] is a highly valuable area of research.

With the rapid advancement of computer vision technology, researchers have increasingly adopted deep learning techniques for animal behavior recognition. Man et al. [10] used YOLOv5 to identify four behaviors of sheep—lying, standing, eating, and drinking—achieving an accuracy of 96.7%. Yalei et al. [11] developed a hybrid network structure combining YOLO and LSTM, which enabled the identification of aggressive behaviors in groups of sheep, achieving a precision of 93.38%. Hongke et al. [12] proposed a high-performance sheep image instance segmentation method based on the Mask R-CNN framework, achieving box AP, mask AP, and boundary AP scores of 89.1%, 91.3%, and 79.5%, respectively, on the test set. Zishuo et al. [13] introduced a two-stage detection method based on YOLO and VGG networks, which achieved behavior recognition of sheep with a classification accuracy exceeding 94%.

In the field of cattle behavior recognition, various behavior recognition models derived from the YOLO algorithm have been widely applied. These models have achieved recognition accuracies exceeding 94.3% in identifying conventional behaviors like drinking, ruminating, walking, standing, lying down, eating, and estrus behaviors [14,15,16]. Cheng et al. [17] proposed a method for herd behavior recognition based on a dual attention mechanism, integrating improved Squeeze-and-Excitation (SE) and Convolutional Block Attention Module (CBAM) attention modules with the MobileNet network, achieving an accuracy of up to 95.17%.

For other animal behavior research fields, such as research into pigs and dogs, researchers have utilized Convolutional Neural Networks (CNNs), Long Short-Term Memory (LSTM) networks, DeepSORT networks, YOLOX series networks, ResNet50, and PointNet, among others. By designing improved models based on optimized parameters or multi-network fusion models with enhanced network structures, researchers have achieved recognition of base behaviors (walking, standing, and lying) [18,19], individual identification [20], eating time statistics [21], 3D posture estimation [22,23], emotion recognition [24], and aggression detection [25], with accuracy rates consistently exceeding 90%.

In recent years, researchers have applied 3D convolutional networks and dual-stream convolutional networks to the field of animal behavior recognition. Kaifeng et al. [26] introduced a dual-stream convolutional network for pig video behavior recognition, which combines Inflated 3D Convolutional Network (I3D) and Temporal Segment Network (TSN), achieving an average recognition accuracy of 98.99% for behaviors such as eating, lying down, walking, scratching, and mounting. Yunfei et al. [27] proposed an E3D (Efficient 3D CNN) algorithm for the accurate and rapid recognition of basic dairy cow behaviors (lying, standing, walking, drinking, and feeding). The precision, recall, parameters, and FLOPs of the E3D were 98.17%, 97.08%, 2.35 M, and 0.98 G, respectively. The E3D algorithm achieves the recognition of basic cow behaviors in video data with fewer parameters and computational requirements. Bo et al. [28] proposed a method that combines the SlowFast algorithm with the Hidden Markov Model (HMM) to recognize sow nursing behaviors (before piglet sucking, piglet sucking, and end of piglet sucking). The study indicated that the SlowFast model exhibits the best overall performance in fine-grained sow nursing behavior classification. When combined with HMM, the approach effectively performs fine-grained sow nursing recognition tasks, achieving sequence overlap and behavior transition time localization accuracy rates of 90.51% and 87.05%, respectively. Similarly, Gang et al. [29] selected the 3D ResNet50 network as the backbone of the SlowFast dual-path framework and proposed an improved SlowFast algorithm for recognizing basic yak behaviors. By increasing the size of the 3D convolution kernel, the perceptual field of feature extraction is improved, thus effectively improving the recognition accuracy of the algorithm. The method achieved classification of basic yak behaviors in natural scenes with a recognition accuracy of 96.6%. This research lays the foundation for real-time monitoring of yak health status on the Tibetan Plateau.

In summary, researchers have achieved significant advancements using various smart devices and advanced algorithms in their respective fields. However, in the field of intelligent recognition of horse behaviors, there is no relevant research. This study analyzes the methodologies proposed by other researchers and adopts the SlowFast algorithm [30] as the foundational approach to recognize horse behaviors, focusing on the daily activities of horses in stalls. The chosen algorithm enables multi-label recognition and annotates the identified behaviors with bounding boxes, making the recognition results more intuitive and practically applicable. The contributions of this study are as follows:

(1) Developed an AVA-format dataset specifically for horse behavior recognition, encompassing five categories: standing, sternal recumbency, lateral recumbency, sleeping, and eating.
(2) Integrated a Squeeze-and-Excitation (SE) attention module into the SlowFast network and proposed an improved loss function, which enhances the accuracy of horse behavior recognition using the SlowFast network.
(3) Incorporated YOLOX into the SlowFast network, increasing the efficiency of recognizing horse targets in video data.

## 2. Materials and Methods

### 2.1. Label Definition of Horse Posture and Behavior

This study distinguishes between horse postures and behaviors, which is common knowledge in horse behavior research. The postures of horses include standing, lying in sternal recumbency, and lying in lateral recumbency. The eating behavior of horses refers to when they lower their heads to eat food from the ground while in a standing posture. The sleeping behavior of horses includes wakefulness, drowsiness, slow-wave sleep, and paradoxical sleep. To better achieve automatic recognition of horse behaviors, as shown in Table 1, we define the category labels used to annotate horse behaviors in this study. These labels include three posture categories: standing, sternal recumbency, and lateral recumbency, as well as two behavior categories: eating and sleeping.

### 2.2. Experiment and Data Collection

**Figure 1:**
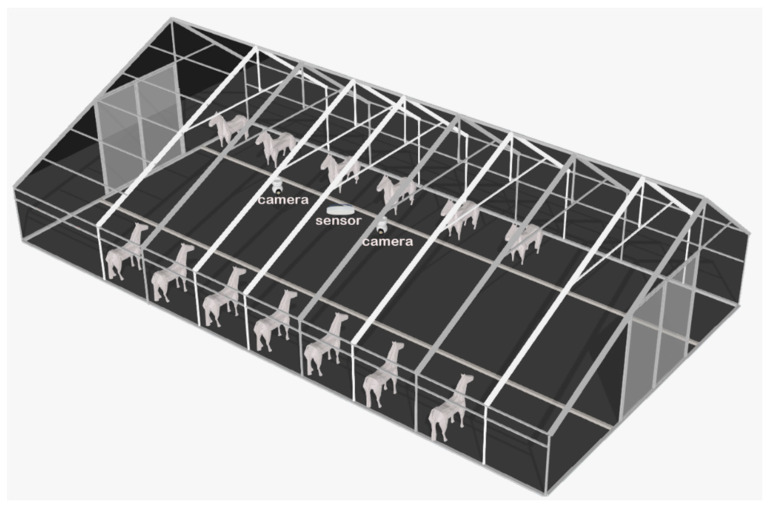
Schematic diagram of data collection scenario.

The subjects of this study are horses kept in individual stalls at an experimental station (Xinjiang Ancient Ecological Park Akhal-Teke Horse Base in Urumqi, Xinjiang Uygur Autonomous Region, China). The data collection period went from May to August 2023. To avoid disturbing the horses, this study used high-definition smart camera equipment for data collection. As shown in Figure 1, the cameras were installed on the crossbeam of the stall. The resolution of the captured video data was 1920 × 1080 pixels, with a frame rate of 30.0 frames per second. A total of 12 horses were recorded, and due to reasons such as horses being outside the stable or power outages in the camera equipment, the video durations range from 10 min to 2 h. Excluding instances where the horses were not present in the stall, the dataset comprised 3.48 TB (Terabytes) of horse behavior video data.

### 2.3. Dataset Construction

The establishment of the horse behavior dataset is as follows: (1) The dataset must include data from different time periods throughout the data collection cycle to ensure the robustness of the model. (2) The dataset includes 12 horses ranging from 7 to 14 years of age. The inclusion of horses from different age stages enhances the adaptability of the model. (3) The dataset must include the main horse breeds from the experimental base to ensure sample diversity. We excluded any video data where horse behaviors were not clearly visible under low light conditions, horses moved outside the camera’s range, or horses were not entirely captured within the frame. Based on these criteria, we compiled a video subset representing the original horse behavior data, comprising a total of 32 video segments.

As shown in Table 2, horse postures include standing, sternal recumbency, and lateral recumbency, while horse behaviors include sleeping and eating. Each video segment is 60 s, with a total duration of 1920 s. Additionally, the number of clips for each category is shown in Table 2.

**Figure 2:**
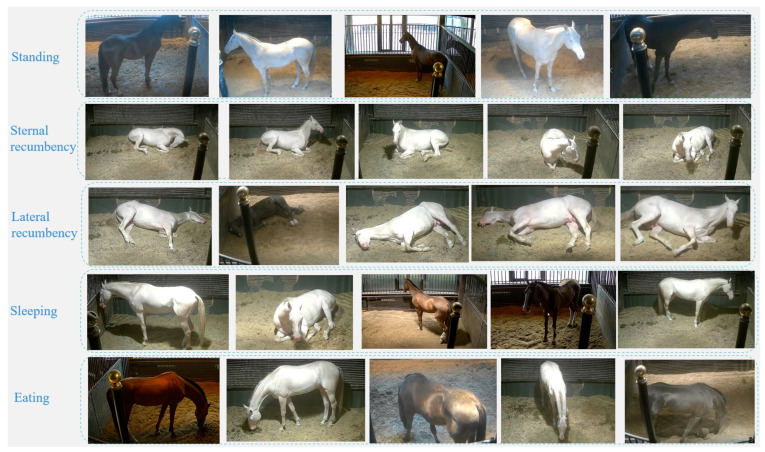
Dataset samples.

In accordance with the AVA (Aesthetic Visual Analysis) dataset format requirements, two methods were used for frame extraction from the video data: extracting 1 frame per second and extracting 30 frames per second. A total of 59,520 frames were extracted. Among these, frames extracted at a rate of 1 frame per second were labeled, resulting in a total of 1920 labeled images. The dataset was divided into training and testing sets in an 8:2 ratio, with 1536 labeled images in the training set and 384 labeled images in the testing set. Figure 2 displays some samples from the dataset, which include various postures and behaviors of different adult horses under different lighting conditions.

### 2.4. Data Enhancement

**Figure 3:**
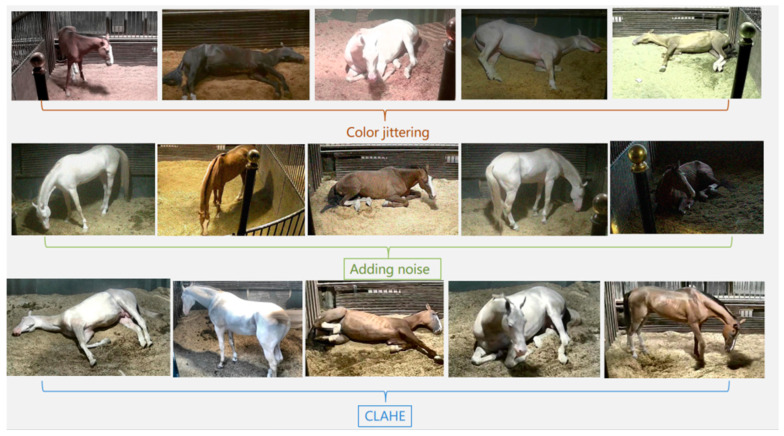
Example of data enhancement.

To achieve a model with better performance and robustness, as shown in Figure 3, we randomly selected a subset of images from the dataset for data enhancement. Three methods were employed: Color Jittering, Adding Noise, and CLAHE (Contrast Limited Adaptive Histogram Equalization). Color Jittering enhances the model’s robustness against variations in lighting and color changes. Adding Noise effectively improves the model’s resistance to noisy environments. CLAHE enhances the model’s ability to process images with uneven lighting conditions.

### 2.5. Overall Technical Route

**Figure 4:**
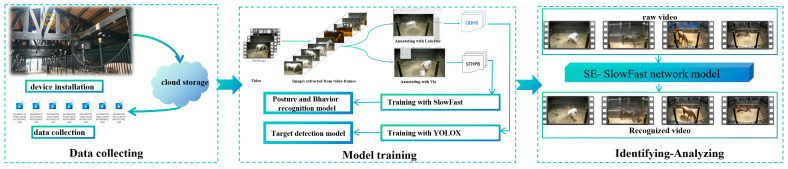
Overall technical route.

The overall technical route used in this study is illustrated in Figure 4, which is divided into three stages: data collection, model training, and identifying-analyzing. In the data collection stage, high-definition cameras were deployed on the beams of the stall to collect video data of the horses. The collected video data were stored on a cloud server. In the model training stage, we constructed two datasets using a set of identical frame images: a COCO (Common Objects in Context)-format dataset for YOLOX target detection, named ODHS (Object Detection Dataset of Horses in Stall), and a spatio-temporal dataset in AVA format for SE-SlowFast behavior recognition, referred to as STHPB (Spatio-Temporal Dataset of Horse Postures and Behaviors). The SE-SlowFast model was trained on the STHPB dataset to obtain a pre-trained model for horse behavior recognition, which can be used to identify horse behaviors in video frame sequences. Additionally, YOLOX was used to replace the original object detector, FastRCNN, in the SlowFast algorithm. This replacement improves both the speed and accuracy of detecting horse targets in videos. In the final stage, we performed behavior recognition on the horse video data, comprehensively recording and analyzing the recognition results.

In addition, the hardware environment used in the experiment consisted of a server equipped with a 24 GB NVIDIA RTX 3090 GPU, 60 GB of RAM, a 1 TB hard drive, and an AMD EPYC 9754 128-core processor CPU. The operating system was Ubuntu 20.04, and the algorithms were developed using the PyTorch 3.8 deep learning framework and the Python programming language.

### 2.6. Model Implementation

#### 2.6.1. SE-SlowFast Network

**Figure 5:**
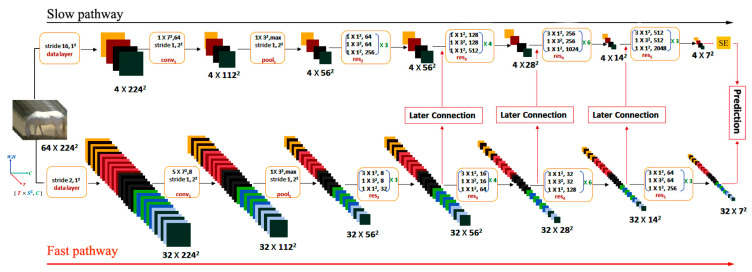
The architecture of spatiotemporal convolutional network for horse posture and behavior recognition: The backbone network uses ResNet50, and the dimension size of the kernel is $\left\{T\times S^{2},C\right\}$, where T represents the time dimension size, $S^{2}$ represents the spatial dimension size, and C represents the channel size.

This study is based on the dual-stream architecture of the SlowFast network. As shown in Figure 5, the SE attention module is added at the end of the slow pathway. The improved SlowFast network consists of three main components: the slow pathway with the added SE attention module, the fast pathway, and the later connections.

##### SlowFast Network

The SlowFast network comprises a Slow Pathway and a Fast Pathway; both the Slow Pathway and Fast Pathway use ResNet50 as the backbone network. As shown in Formula (1), the Slow Pathway processes long temporal sequences at a lower frame rate. The input frame length is set as $L_{slow}$ = 64, and the time step size is $S_{slow}$ = 16, effectively addressing the issue of temporal downsampling. $N_{slow}$ = 4, effectively addressing the issue of temporal downsampling.

(1) $$N_{slow}=\frac{L_{slow}}{S_{slow}}$$

Compared to the Slow Pathway, the Fast Pathway adopts a time step of 2, through similar calculations, the number of sampled frames $N_{fast}\;$= 32. As shown in Formula (2), $C_{slow}$ is the number of channels for the Slow Pathway, set $C_{slow}\;$= 64. The number of channels for the Fast Pathway is calculated to be $C_{fast}\;$= 8. This configuration ensures that the Fast Pathway exhibits superior accuracy.

(2) $$C_{fast}=\frac{1}{8}C_{slow}$$

After feature matching is completed, lateral connections link the Fast Pathway to the Slow Pathway. Through multiple lateral connections, the SlowFast network achieves the fusion of feature information from both stream branches. Finally, the fused feature information is fed into a classifier for predicting equine behavior classification.

##### SE Module

**Figure 6:**
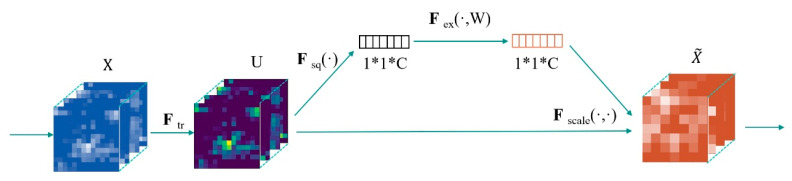
Structure diagram of SE Module.

The basic structure of the SE Module is shown in Figure 6. For any given transform $F_{tr}$: X → U, X ∈ R^W′×H′×C′^, U ∈ R^W×H×C^. $F_{tr}$ is a standard convolutional operator process. The symbol * represents the multiplication operation, used to describe the spatial dimensions. As shown in Formula (3), X represents the input feature map and $V_{c,c^{\prime }}$ is the convolution kernel. The symbol ∗ denotes the convolution operation. Through this convolution operation, each layer of the input feature map X undergoes a convolution operation with a 2D convolution kernel, ultimately producing C output feature maps, which form the feature map U.

(3) $$U_{C}=F_{tr}\left(X\right)=\sum_{c^{\prime }=1}^{C^{\prime }}\left(X_{c^{\prime }}*V_{c,c^{\prime }}\right)$$

After obtaining U, the spatial information is compressed into a channel using the descriptor F*_sq_*, which results in a feature vector. $F_{ex}$ is implemented using a two-layer fully connected network, combined with ReLU and Sigmoid activation functions to perform excitation operations, resulting in a feature vector with channel information. As shown in Formula (4), C is the number of channels, and $s_{c}$ is the excitation weight of channel C. z is the compressed feature vector, and W × H is the spatial dimension of U. σ is the Sigmoid function, and δ is the ReLU activation function. $W_{1}$ and $W_{2}$ are the weight matrices of the two fully connected layers. Through this calculation, the feature map with global information, W × H × C, is directly compressed into a 1 × 1 × C feature vector S containing channel information.

(4) $$s_{c}=F_{sq}\left(U\right)\cdot F_{ex}\left(z\right)=\frac{1}{W\times H}\sum_{j=1}^{W}\sum_{k=1}^{H}U_{j,k,c}\cdot \sigma (W_{2}\cdot \delta (W_{1}z))$$

Finally, the output $\tilde{X}\;$ of the SE module is obtained through re-weighting, where $F_{scale}$ represents this process. As shown in Formula (5), the attention weights $s_{c}$ are applied to each channel ${\;U}_{C}$, and through this operation, the final output is derived.

(5) $${\tilde{X}}_{c}=F_{scale}\left(U_{C},s_{c}\right)=s_{c}\cdot U_{C}$$

#### 2.6.2. YOLOX Network

**Figure 7:**
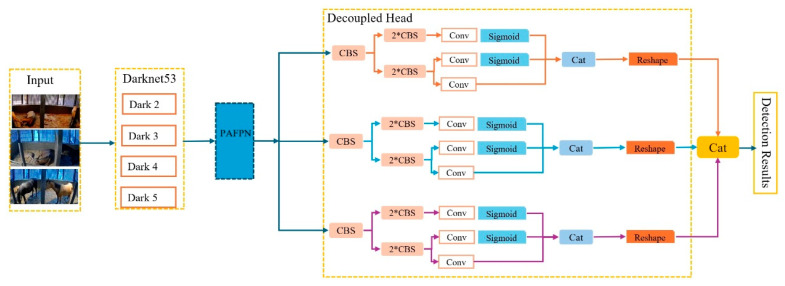
Structural diagram of YOLOX.

To better recognize equine targets in key frames of the video, we utilized YOLOX as the object detection algorithm, as illustrated in Figure 7. YOLOX is based on YOLOV3 and Darknet 53, employing the structure of the Darknet 53 backbone network and SPP layer. A key feature of YOLOX is the use of a Decoupled Head, which enhances the convergence speed of the model and achieves end-to-end performance.

### 2.7. Improved Loss Function

In classification tasks, the commonly used loss function for multilabel classification is Binary Cross Entropy Loss (BCE Loss). However, BCE Loss may not perform optimally when dealing with imbalanced datasets, leading to lower accuracy during model training. Therefore, this study addresses the characteristics of the STHPB dataset and adopts the CW Loss (Class Weighted Loss) combined with Focal Loss to form the CW_F_Combined Loss. This aims to reduce the weight of the loss function for categories with more instances and increase the weight for categories with fewer instances in the multilabel dataset. The formula for CW Loss is as follows:

(6) $$L\left(y_{i},{\hat{y}}_{i},w_{1}\right)=-\frac{1}{N}\sum_{i=1}^{N}[w_{i}*(y_{i}*\log\left(\sigma ({\hat{y}}_{i})\right)+\left(1-y_{i}\right)*log(1-\sigma ({\hat{y}}_{i})))]$$

(7) $$W_{i}=\frac{\sum_{j=1}^{N}y_{j}}{N*y_{ji}},for\;i=1,2,\ldots ,C$$

(8) $$Focal\_Loss=r*{\left(1-p_{t}\right)}^{r}*L,r=2,3\ldots$$

Formula (6) is the definition of the CW Loss function. In the formulas, N represents the sample quantity, σ is the sigmoid function, ${\hat{y}}_{i}$ denotes the predicted score of the model, $y_{i}$ signifies the true label, and $w_{i}$ represents the class weight. Formula (7) is the definition of the class weight calculation formula. C is the total number of classes. The CW Loss function consists of two main parts: the first part calculates the confidence of positive classes, and the second part calculates the confidence of negative classes. When $y_{i}\;$ = 1, the first part predominates, maximizing the model’s confidence in positive classes; when $y_{i\;}$ = 0, the second part predominates, maximizing the model’s confidence in negative classes. The purpose of $w_{i}$ is to multiply the loss terms for each sample by the corresponding category weight during the computation of the loss function. This ensures that categories with fewer instances contribute more significantly to the loss during training. Formula (8) is the definition of Focal_Loss, Passing the value of $L\left(y_{i},{\hat{y}}_{i},w_{1}\right)$ to Focal_Loss, and the final loss function is computed, effectively addressing the issue of data imbalance.

### 2.8. Model Evaluation Metrics

To objectively analyze the model’s performance, this study adopts the Mean Average Precision (mAP) with IoU = 0.5 as an evaluation metric. The formula is as follows:

(9) $$IoU\left(A,B\right)=\left|\frac{A\cap B}{A\cup B}\right|$$

(10) $$mAP=\int_{0}^{1}P\left(R\right)dR$$

(11) $$R=\frac{T_{rue}P_{ositive}}{T_{rue}P_{ositive}+F_{alse}N_{egative}}$$

(12) $$P=\frac{T_{rue}P_{ositive}}{T_{rue}P_{ositive}+F_{alse}P_{ositive}}$$

Formula (9) represents the Intersection Over Union (IoU) threshold calculation, where A denotes the ground truth bounding box and B represents the predicted detection box. Formula (10) is the calculation formula for mAP, where R is recall and P is precision. Formulas (11) and (12) provide the calculation formulas for R and P, $T_{rue}P_{ositive}$ represent the number of samples correctly predicted as positive by the model, $F_{alse}N_{egative}$ denote the number of positive samples incorrectly predicted as negative, and $F_{alse}P_{ositive}$ represent the number of negative samples incorrectly predicted as positive.

## 3. Experiments and Results

### 3.1. Horse Object Detection

**Figure 8:**
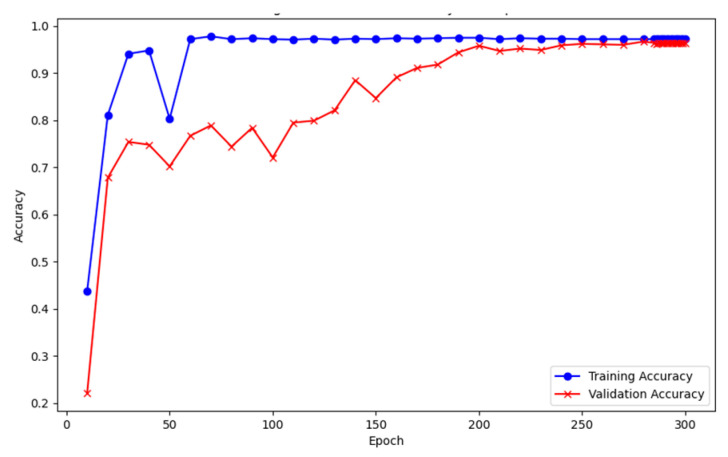
The accuracy of YOLOX training.

To ensure that the SE-SlowFast algorithm can efficiently recognize horse behaviors in video data, we labeled the extracted frame images to create a target detection dataset, ODHS (Object Detection Dataset of Horse in Stall), with a total of 1920 labeled images. The category label is “Horse”, and the dataset is divided into training and test sets in a ratio of 8:2. YOLOX was utilized for object detection training, and, as shown in Figure 8, The blue curve represents the accuracy of the training set, while the red curve represents the accuracy of the validation set. When Epoch = 60, the accuracy of the training set begins to stabilize, with the optimal accuracy reaching 97.2%. In comparison, as the number of training epochs increases, the accuracy of the validation set stabilizes at Epoch = 200, with the optimal accuracy reaching 96.4%. This accuracy ensures that YOLOX, integrated with the SE-SlowFast network, can effectively detect the horse targets during the video data recognition phase.

**Figure 9:**
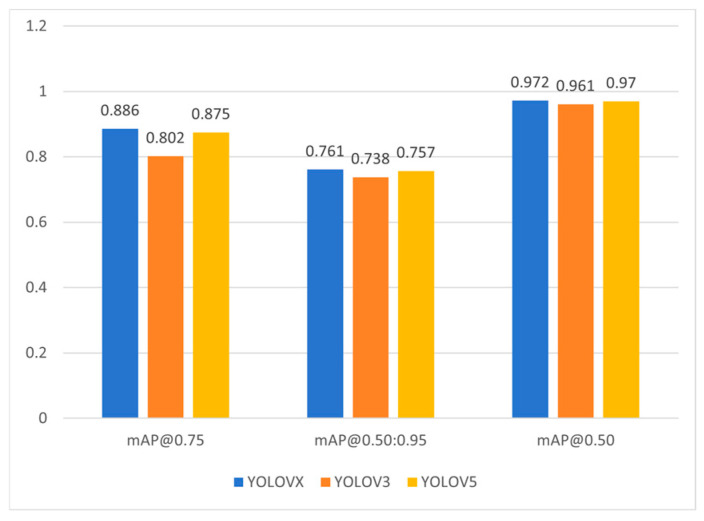
YOLOX vs. other versions of YOLO.

This study compares the performance of YOLOX with other versions of the YOLO algorithm on the ODHS, such as YOLOV3 and YOLOV5. During the training process, the network structures of the three models were kept unchanged, and the training parameters were ensured to be consistent. The final training results are shown in Figure 9. The results indicate that, although YOLOV3 and YOLOV5 have unique network structures, their performance on this dataset was not satisfactory. Therefore, we ultimately selected YOLOX.

### 3.2. Behavior Recognition of Horses

#### 3.2.1. Feature Learning Effect of SE-SlowFast Network

**Figure 10:**
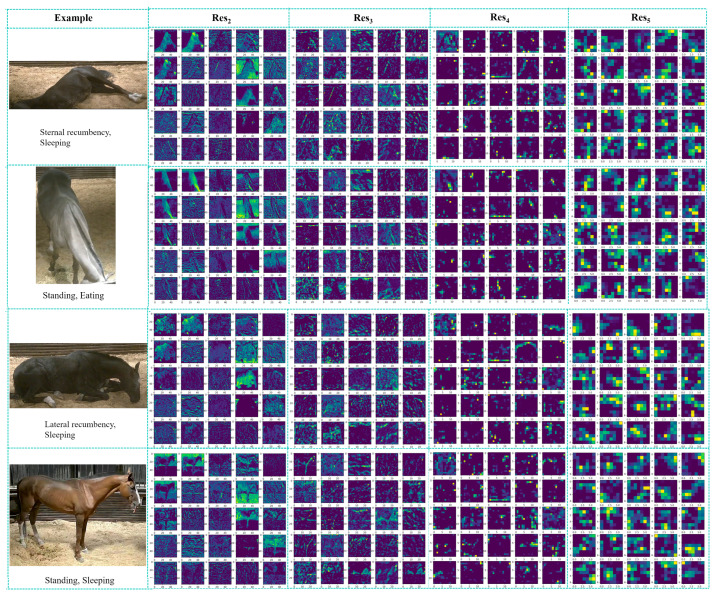
Example of Slow pathway Feature Learning: Res_2_, Res_3_, Res_4_, Res_5_ correspond to Figure 5. Each feature map learned after the convolution operation has sizes: 56^2^, 28^2^, 14^2^, and 7^2^.

In the SlowFast algorithm, the Slow pathway primarily handles behavioral feature extraction, while the Fast pathway focuses on capturing dynamic information to compensate for the Slow pathway’s limitations in this area. By integrating the Slow and Fast pathways in a coordinated manner, the SlowFast network can simultaneously capture both slow and fast dynamic information in videos, enhancing its ability to recognize actions effectively. In the experiment, we visualized the output features of the Slow pathway. As shown in Figure 10, the visualizations of feature extraction were performed before each of the later connections, resulting in a total of four extractions. It can be observed that, before the first later connection, the Slow pathway had already learned behavioral features from the input image. For instance, in the ‘Standing, eating’ image, due to the horse’s uniquely long neck, we can clearly observe that the Slow pathway has effectively learned the main features of the image during the initial feature extraction. Similarly, after the first feature extraction, it is evident that the main features of the other three images have also been learned by the Slow pathway. As the network deepens, feature learning becomes more sophisticated; by the third and fourth rounds of feature extraction, it becomes challenging for human observers to interpret these deeper features accurately. However, with close observation, the Slow pathway appears to have learned the features of the ‘Standing, Sleeping’ image even in the second feature extraction. Through these feature maps, it is evident that the SlowFast network has successfully learned key behavioral features of the horse.

#### 3.2.2. Comparison of Loss Functions

**Figure 11:**
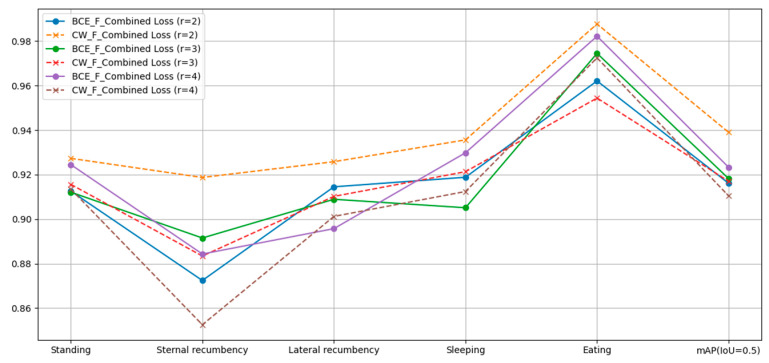
Model performance comparison under different loss functions.

This study compares the training performance of BCE_F_Combined Loss (Binary CrossEntropy Loss combined with Focal Loss) and CW_F_Combined Loss (Class Weighted Loss combined with Focal Loss). In the experiments, we found that when r < 2 or r > 4, the accuracy significantly decreased. Therefore, a total of six experimental rounds were conducted, each assigning a different value of r to Focal Loss. As shown in Figure 11, the model’s prediction accuracy varied with different r values. When r = 2, training with CW_F_Combined Loss improved the accuracy across all categories, with a particularly notable increase in the accuracy for ‘Sternal recumbency’, resulting in the highest overall accuracy.

#### 3.2.3. Ablation Experiment

Table 3 shows the results of the ablation experiments. √ indicates that the module is used, while × indicates that the module is not used. When the SlowFast network does not incorporate the SE attention module, the overall accuracy is relatively low, with the highest accuracy being 90.51% for sternal recumbency. Adding the SE attention module at the front of the Slow Pathway, there was no significant improvement in overall accuracy. When the SE attention module was added to the end of the Slow Pathway, the accuracy of the SlowFast network showed a significant improvement. Especially when r = 2, the accuracy for eating reached 98.77%.

### 3.3. Recognition Results

**Figure 12:**
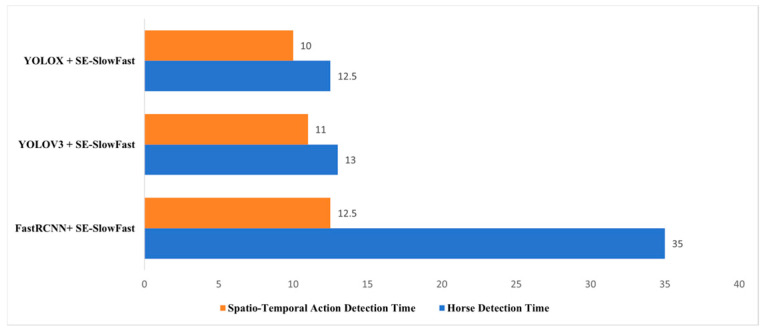
Comparison of different algorithms for video frame detection and Spatio-Temporal Action Detection time.

The SlowFast network relies on a target detection algorithm for behavior recognition, and this study uses YOLOX as the detector. To further evaluate the video recognition efficiency of YOLOX + SE-SlowFast, ten 60 s videos were used in the experiment, with detection time measured in seconds. The horses in the videos were identified first, followed by an analysis of their spatiotemporal behavior. As shown in Figure 12, when comparing the horse detection time and spatiotemporal movement detection time, both YOLOV3 + SE-SlowFast and YOLOX + SE-SlowFast significantly improved video recognition processing times compared to FastCNN + SE-SlowFast. YOLOX + SE-SlowFast demonstrated the best processing speed and faster overall performance. Therefore, YOLOX can serve as an effective target detection algorithm for horses, combined with the SE-SlowFast algorithm to achieve rapid recognition of horse behaviors.

**Figure 13:**
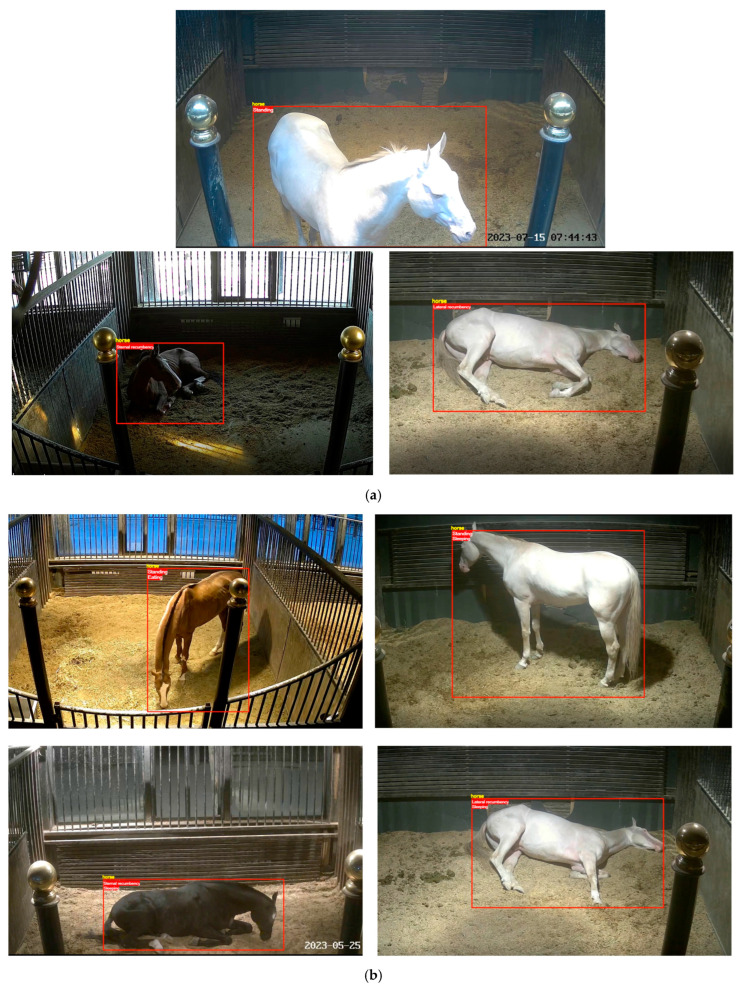
Examples of predicting horse postures and behaviors. (**a**) Predictions of horse postures. (**b**) Predictions of horse behaviors.

The video detection results are shown in Figure 13. (a) shows the recognition results of horse postures, accurately identifying three postures: standing, sternal recumbency, and lateral recumbency. (b) shows the recognition results of horse behaviors, accurately identifying the eating and sleeping behaviors of horses in different postures.

## 4. Discussion

### 4.1. Analysis of Misjudgments and Missed Detections in Horse Behavior Recognition

**Figure 14:**
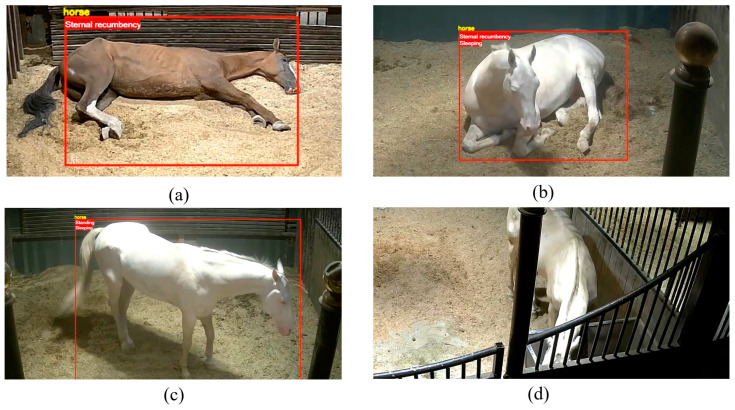
Examples of misjudged and missed detections. (**a**–**c**) is misjudged, (**d**) is missed detections.

This study identified three postures of horses: standing, sternal recumbency and llateral recumbency, and two behaviors: eating and sleeping, achieving high accuracy rates for recognizing postures and behaviors. However, during video recognition, instances of misjudgment and missed detections occurred. As shown in Figure 14, (a) illustrates a misjudgment where the lateral recumbency was incorrectly identified as sternal recumbency. This misjudgment may have resulted from the color of the horse’s legs and hooves being similar to the surrounding environment, making the features of the legs and hooves less distinct. (b) and (c) show instances where the sleeping behavior was misidentified while the horse was in sternal recumbency and standing postures. At these times, the horse was not actually sleeping, as significant movements of its legs and tail were observed. A review of the video confirmed that the horse was indeed awake. Determining sleeping behavior is a challenge in this study. Labeling sleeping behavior under the guidance of animal researchers is a particularly challenging task, as horses may sleep with their eyes open or closed, and even when sleeping standing up, they may make slight leg movements to relieve fatigue. Achieving more accurate labeling of sleeping behavior during the dataset annotation phase could be one potential solution to address misjudgments. (d) displays instances where no behavior was recognized. This may indicate a lack of similar target detection images in the Object Detection Dataset of Horse in Stall (ODHS). Since target detection is a prerequisite for the SlowFast network to perform behavior recognition, expanding the ODHS as much as possible is one of the methods to resolve missed detections.

### 4.2. The Connection Between Horse Basic Behavior Recognition and Horse Health

This study focused only on the basic postures and behaviors of horses in the experimental stall, which can provide references for assessing the horses’ health conditions. For instance, when a horse’s legs and hooves are uncomfortable, the duration of standing significantly decreases, while the duration of lateral recumbency correspondingly increases. Horses are not ruminants, but like ruminants, the duration and frequency of their eating behavior can directly reflect their health condition. Sleeping behavior is closely related to the health of horses. Thus, identifying and studying the basic behaviors of horses provides a convenient method for early awareness of their health status. Additionally, this study lays the foundation for the identification of other advanced behaviors and intelligent perception of horse health.

### 4.3. Follow-Up Research Directions for Horse Behavior Recognition

(1) Unlike human behavior recognition, achieving more refined recognition of horse behaviors is a challenging task, such as accurately distinguishing between drowsiness, slow wave sleep (SWS), and paradoxical sleep. Therefore, in future research, closer collaboration between computer vision specialists and animal behavior scientists is essential to enable more precise intelligent recognition of horse behaviors. (2) The recognition of individual horses’ behaviors in the stall has not been linked to their daily behaviors outdoors, which prevents us from associating the behavior recognition results with the daily activities of specific horses throughout the day. Hence, accurately identifying individual horses and establishing connections between their behaviors and an analysis of their daily activities will be a focal point of future research. (3) The duration and frequency of basic horse postures and behaviors are important indicators of health status. Establishing standards for changes in the duration and frequency of basic postures and behaviors when horses are unwell is crucial for monitoring and predicting their health. This will aid in the early detection and treatment of sick horses, thereby benefiting their overall health.

## 5. Conclusions

By accurately recognizing the basic behaviors of horses in stalls using computer vision technology, horse breeding enterprises and equestrian club managers can effectively achieve intelligent supervision, save human resources, and improve the health conditions of horses. Additionally, this approach benefits the breeding of rare horse breeds. This study uses the SE-SlowFast algorithm to achieve spatiotemporal feature recognition of horse postures and behaviors in video data. The method effectively distinguishes key postures of horses, such as standing, lying in sternal recumbency, and lying in lateral recumbency, as well as recognizing behaviors like sleeping and eating. The method has a wide range of applications and can accurately identify the behaviors of horses in videos. Therefore, this study provides valuable references for the advanced behavior recognition of multiple horses and holds significant importance for the physiological health assessment of horses in the context of intelligent precision breeding, laying the foundation for the sustainable development of equine management.

## Figures and Tables

**Table 1:** Label definitions for horse posture and behavior.

Label	Posture/Behavior	Description
Standing	Posture	All 4 hooves of the horse touch ground, supporting the horse's body.
Sternal recumbency	Posture	Lying in sternal recumbency, the horse is lying on its chest with all four legs stretched out to one side.
Lateral recumbency	Posture	Lying in lateral recumbency, the horse is lying flat on the side, with head and legs touching the ground.
Eating	Behavior	The horse lowers its head to eat food on the ground.
Sleeping	Behavior	Sleep includes Drowsiness, Slow Wave Sleep (SWS), and Paradoxical Sleep. Horses in these sleep states may be standing, lying in sternal recumbency, or lying in lateral recumbency, with their eyes either open or closed.

**Table 2:** Horse behaviors data.

Postures and Behaviors	Number of Videos	Video Duration (s)	Number of Frame Images
Standing	3	60	5580
Standing, Eating	11	60	20,460
Standing, Sleeping	4	60	7440
Sternal recumbency	4	60	7440
Sternal recumbency, Sleeping	3	60	5580
Lateral recumbency	3	60	5580
Lateral recumbency, Sleeping	4	60	7440

**Table 3:** Results of ablation experiments.

Backbone Network	SE Module at the Front of the Slow Pathway	SE Module at the End of the Slow Pathway	CW_F_Combined Loss			Accuracy of Postures and Behaviors Recognition				
			r = 2	r = 3	r = 4	Standing	Sternal Recumbency	Lateral Recumbency	Sleeping	Eating
SlowFast	×	×	√			0.8945	0.9051	0.8829	0.9011	0.8947
	×	×		√		0.8705	0.8624	0.8744	0.9198	0.9029
	×	×			√	0.8691	0.8369	0.8601	0.9035	0.9172
	√	×	√			0.9023	0.9122	0.8754	0.9123	0.9189
	√	×		√		0.9048	0.8793	0.8597	0.8945	0.8803
	√	×			√	0.8958	0.8382	0.8447	0.8795	0.9108
	×	√	√			0.9273	0.9187	0.9258	0.9356	0.9877
	×	√		√		0.9156	0.8834	0.9102	0.9213	0.9544
	×	√			√	0.9135	0.8525	0.9012	0.9124	0.9725

## Data Availability

Data are available on request from the authors.
